# Value and Limits of Routine Histology Alone or Combined with Glutamine Synthetase Immunostaining in the Diagnosis of Hepatocellular Adenoma Subtypes on Surgical Specimens

**DOI:** 10.1155/2013/417323

**Published:** 2013-02-19

**Authors:** Paulette Bioulac-Sage, Saïd Taouji, Brigitte Le Bail, Laurent Possenti, Charles Balabaud

**Affiliations:** ^1^Service d'Anatomie Pathologique, Hôpital Pellegrin, CHU Bordeaux, Place Amélie Raba Léon, 33075 Bordeaux Cedex, France; ^2^Inserm U1053 Université Bordeaux Segalen, 146 rue Leo Saignat, 33076 Bordeaux Cedex, France; ^3^Hôpital St. André, CHU Bordeaux, 33000 Bordeaux Cedex, France

## Abstract

Immunohistochemistry is a valid method to classify hepatocellular adenoma (HCA). The aim was to test the performance of routine histology combined to glutamine synthetase (GS) staining to identify the 2 major HCA subtypes: HNF1**α** inactivated (H-HCA) and inflammatory HCA (IHCA). 114 surgical cases, previously classified by immunohistochemistry, were analysed. Group A comprised 45 H-HCAs, 44 IHCAs, and 9 **β**-catenin-activated IHCAs (b-IHCA), and group B, 16 b-HCA and unclassified HCA (UHCA). Steatosis was the hallmark of H-HCA. IHCA and b-IHCA were mainly characterized by inflammation, thick arteries, and sinusoidal dilatation; b-IHCA could not be differentiated from IHCA by routine histology. Group B was identified by default. A control set (91 cases) was analyzed using routine and GS stainings (without knowing immunohistochemical results). Among the 45 H-HCAs and 27 IHCAs, 40 and 24 were correctly classified, respectively. Among the 10 b-IHCAs, 4 were identified as such using additional GS. Eight of the 9 HCAs that were neither H-HCA nor IHCA were correctly classified. *Conclusion*. Routine histology allows to diagnose >85% of the 2 major HCA subtypes. GS is essential to identify b-HCA. This study demonstrates that a “palliative” diagnostic approach can be proposed, when the panel of specific antibodies is not available.

## 1. Introduction

Hepatocellular adenomas (HCAs) are rare benign tumors. Molecular data have brought new insight in the characterization of this disease. They allow the distinction of focal nodular hyperplasia (FNH) from HCA and the identification of 2 major HCA subtypes which represent more than 80% of all HCAs, namely, HNF1A-mutated HCA (H-HCA, 35–40%) [1], and inflammatory HCA (IHCA, 50–55%) [2]; 10% of IHCAs being also *β*-catenin mutated (b-IHCA). The remaining HCAs are the *β*-catenin-mutated HCA (b-HCA, 10%) [3] and the unclassified HCA (UHCA, which account for less than 10%). 

The immunohistochemical (IHC) classification of HCA subtypes was derived from the above-mentioned molecular characterization and showed a good correlation with the molecular data [2]. Using specific IHC markers, such as liver fatty acid-binding protein (LFAPB), C reactive protein (CRP) or serum amyloid A (SAA), glutamine synthetase (GS), and *β*-catenin, it is possible to identify all HCA subgroups with good confidence. Among these markers, GS is of major importance to identify patients at high risk of malignant transformation. Indeed, abnormal GS staining [4] is a strong argument to suggest *β*-catenin activation. Unfortunately, the rarity of these tumors in routine practice leads to the low availability of the specific IHC markers. To overcome this problem, the possibility to identify the 2 major HCA subtypes using standard histological techniques has not been tested. 

The aim of this study was (1) to test routine histology in the diagnosis of HCA subtypes and (2) to appreciate the contribution of GS combined with routine histology in the diagnosis of HCA subtypes. 

## 2. Materials and Methods

### 2.1. Patients and Tissue Samples

All surgically removed HCA cases were retrieved from our files from January 2000 to November 2011. We excluded cases with specific etiologies such as glycogenosis, male hormone administration (since they are very particular and rare conditions), cases of obvious hepatocellular carcinoma (HCC) possibly related to HCA (but without formal confirmation for a previous HCA) and cases with massive hemorrhage or necrosis (but without sufficient nonnecrotic tissue available).

For all patients (114 cases), the following data were available:

1. clinical data: sex, age, body mass index (BMI), oral contraception (OC), and imaging data (number and size of nodules), as well as liver enzymes (AP, GGT) and CRP in blood,
2. routine stainings on paraffin sections in tissue areas devoid or with minimal necrosis or hemorrhage,
3. IHC stainings. Since 2007, IHC has been performed prospectively; prior this date, it had been performed retrospectively in all cases, according to previous published studies [2]; the HCA classification was based on IHC.

Briefly, taking into account our own experience previously published in pathology textbooks showing the very good correlation between molecular analysis and IHC for H-HCA and IHCA [5, 6]: LFABP negativity in tumor (T) and positivity in nontumoral liver (NTL) was interpreted as H-HCA; SAA or CRP staining positive in T and negative in NTL was interpreted as IHCA (even though SAA/CRP detection could be more or less intense and homogeneous). Once the diagnosis of H-HCA was made, SAA or CRP staining was not mandatory, and once the diagnosis of IHCA was made, LFABP staining was not mandatory in such cases. The expression of GS, a target gene of *β*-catenin, was studied in all cases. GS staining was quoted as negative in the absence of abnormal expression; and in this situation, *β*-catenin staining was not mandatory. GS staining in T always differed from the normal distribution of GS which is limited to a few rows of centrilobular hepatocytes in the NTL. In T, GS could be either totally absent or restricted to the border between T and NTL and/or around some persistent veins within T. Abnormal expression of GS characterized in the whole nodule by a strong positive staining diffuse or patchy is easy to interpret but is more difficult to interpret when the staining is faint or focal, limited to individual or groups of positive hepatocytes irregularly distributed within the tumor. In any case, abnormal GS staining was considered as a strong argument to suggest *β*-catenin activation [3, 4]. In these cases, aberrant *β*-catenin nuclear labelling allowed the final diagnosis of b-HCA or b-IHCA. However, the absence of labelled nuclei on the section did not rule out this diagnosis because the number of labelled nuclei can be heterogeneously distributed, extremely limited, or even absent on the section. When all the specific IHC markers were negative, the HCA was termed UHCA. 

Another cohort of 91 HCAs was analyzed for external validation. It corresponded to cases not included in the above series and concerning cases from our center (prior 2000) or from cases sent for advice. For each case, 2 slides of H&E and GS (including the tumoral and nontumoral part) were available. 

### 2.2. Methods

#### 2.2.1. Identification of Major Pathological Features Using Routine Histological Criteria in 114 HCA Cases Previously Classified by IHC

Cases were divided into 2 groups. Group A included H-HCA cases and IHCA (*β*-catenin and non *β*-catenin activated) cases. Group B included all other cases. For each group and subgroup, we analysed the clinical, imaging, and biological data. Routine stainings (H&E, trichrome) as well as CD34, keratin (K)7 immunostainings of the 2 groups were reviewed (T and NTL) according to a flowchart (see Table 1 in Supplementary Material available online at http://dx.doi.org/10.1155/2013/417323) filled out by an experienced liver pathologist (PBS). Different pathological items were checked such as steatosis, sinusoidal dilatation, congestion, inflammation, thick arteries, ductular reaction. Steatosis and sinusoidal dilatation were graded semiquantitatively as indicated in supplemental Table 1. In the NTL, the liver was considered as normal or steatotic (with or without NASH).

#### 2.2.2. External Validation: Identification of HCA Subtypes from the Cohort (91 Cases) Using Standard and GS Staining

Routine slides from HCA previously classified into subgroups using IHC were analyzed blindly by 2 observers (PBS and CB), without knowing IHC results obtained previously. For each case the following diagnosis based on standard features was: H-HCA, IHCA, and b-IHCA with 3 possibilities: yes, no, and possibly for each. For each case the pathological items had to be filled (supplemental Table 1). If the features were obvious, the diagnosis was certain: H-HCA, IHCA (group A) or another type (group B). The diagnosis of H-HCA was uncertain when the result of the reading was H-HCA “possibly,” IHCA “no;” the same was true for IHCA. IHCA cases could be also *β*-catenin activated if GS staining was abnormal. Abnormal GS staining in HCA without characteristics of H-HCA or IHCA was a strong argument to suggest b-HCA. In exceptional cases, we observed abnormal GS staining in H-HCA. Because of the rather straight criteria defined to categorize cases, disagreement among observers was extremely rare, and when it happens, slides were reviewed and a consensus reached.

#### 2.2.3. Statistics

Disparities between groups according to age, BMI score, size, and the number of nodules were analyzed with unpaired *t*-test. Abnormal liver enzyme and CRP levels in each group were compared using ANOVA test. Fisher's exact test was used to compare different types of pathological abnormalities (steatosis and sinusoidal dilatation). *P* values lower than 0.05 were considered as significant. Kappa statistic was used to measure the level of agreement between the standard histological including GS versus the complete set of immunohistochemical techniques to estimate proportions of HCA subtypes.

## 3. Results

### 3.1. Major Pathological Criteria of the HCA Subtypes Using Routine Histological Criteria (114 Cases)

**Figure 1:**
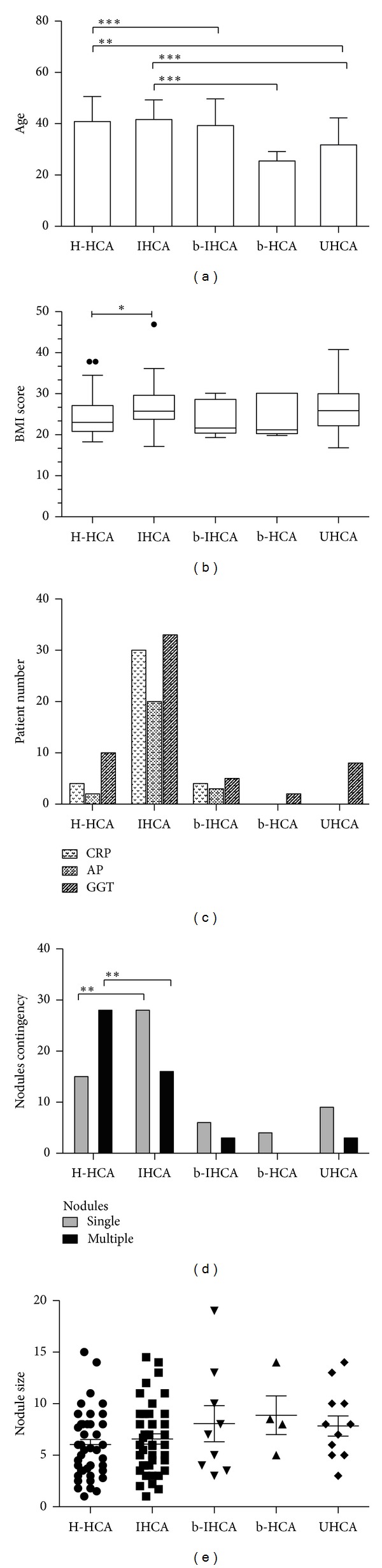
Graphic representation of age (a), BMI score (b), liver enzymes and CRP (c), nodules contingency (d), and nodules size (e) in different HCA subtypes. One asterisk: *P* value < 0.05; two asterisks: *P* value < 0.01; three asterisks: *P* value < 0.001.

**Figure 2:**
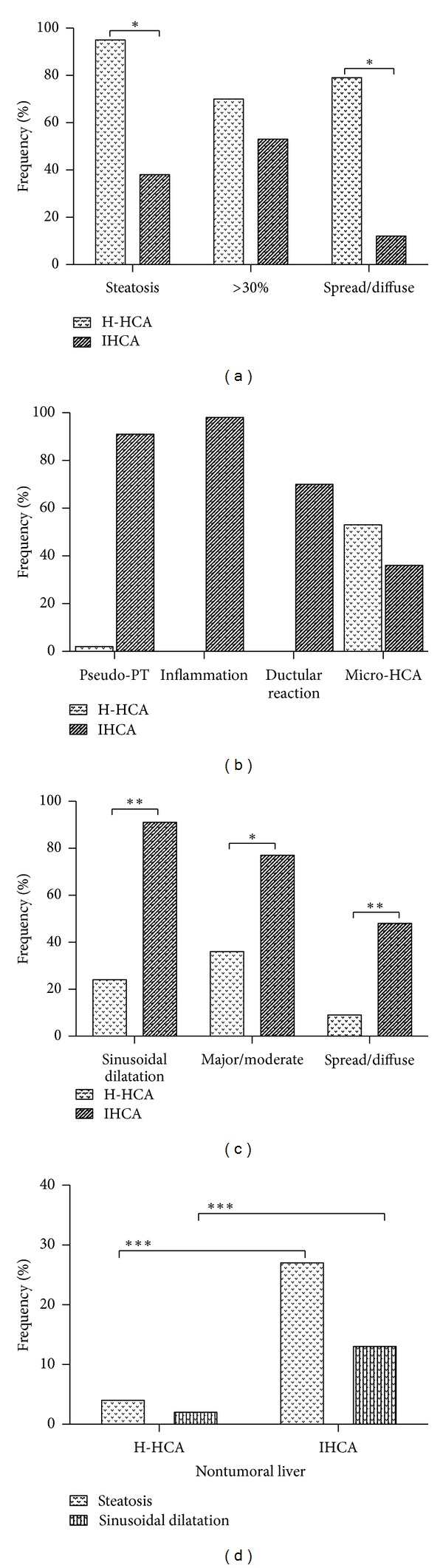
Frequency (%) of various liver pathological abnormalities in the tumoral liver (a–c) and steatosis, sinusoidal dilatation in the non tumoral liver (d) in patients with H-HCA and IHCA. One asterisk: *P* value < 0.05; two asterisks: *P* value < 0.01, three asterisks: *P* value < 0.001.

Amongst the 114 cases analysed by standard morphology and IHC, there were 98 cases in group A: 45 H-HCAs, 53 IHCAs (9 were b-IHCAs) and 16 cases in group B: 4 b-HCAs and 12 UHCAs. The relevant clinical, biological, and pathological data are shown in Figures 1 and 2, Table 1 and supplemental Table 2.

#### 3.1.1. Group A

*H-HCA.* This subgroup included 45 patients, all women; 84.4% were under OC; the median age was 40. The median size of the largest nodule was 5.6 cm, and nodules were multiple in 62.2%. 75% of the patients had normal BMI; CRP and AP were rarely elevated, and 22.2% had mild elevation of GGT. Interestingly enough, in 8 patients, micro/small H-HCA (<1 cm) were incidentally discovered on the surgical specimen removed for large tumor(s) of different types including focal nodular hyperplasia (4 cases), 1 angiomyolipoma, 1 HCC; in other 2 cases, there was a past history of melanoma, and the small nodules were suspected to be metastases. 

**Figure 3:**
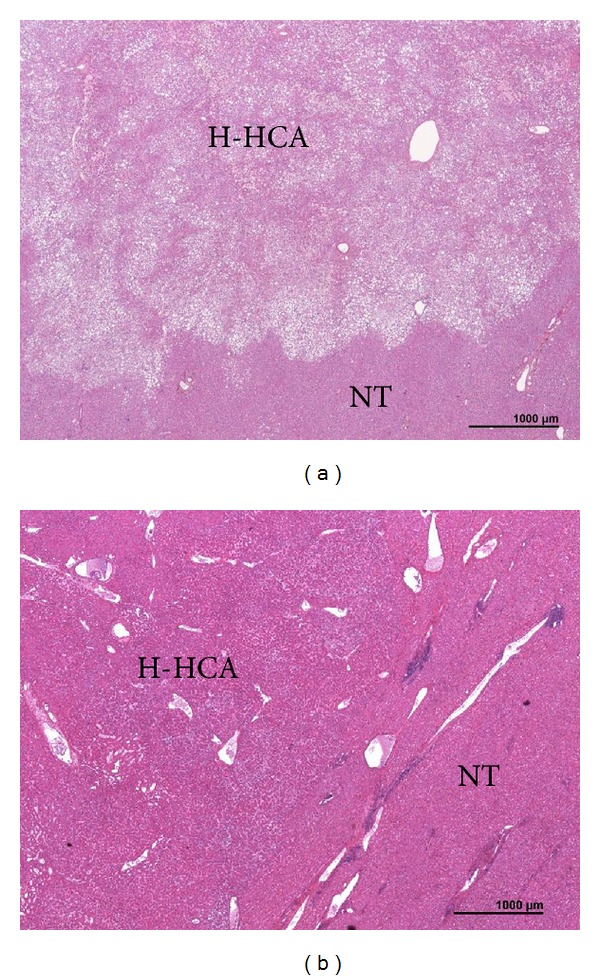
HNF1*α* inactivated HCA (H-HCA)—(a) H&E: typical aspect with diffuse steatosis, thin vessels, and lobulated contours, contrasting with the absence of fat in the nontumoral liver (NT); (b) this nodule of H-HCA does not contain fat, and sinusoids are slightly dilated.

Steatosis was the hallmark of H-HCA, which usually exhibited a very characteristic aspect with lobulated contours (Figures 2(a) and 3(a)). Steatosis was present in 95.6% of cases: severe (>60%), moderate (30–60%), or mild (10–30%) in 25.5, 44.2, and 30.2% respectively; it was totally absent in 2 cases only (Figure 3(b)). Of note, steatosis was spread and/or diffused in 79%. Most of the time, steatotic hepatocytes were intermingled with clear hepatocytes which were predominant in 2 cases, with mild/focal steatosis. Sinusoidal dilatation was present in 24.4% and mild in the great majority of cases. In two cases a few pseudoglandular arrangements were noticed without cytological abnormalities. Micro-H-HCA nodules were observed in surrounding liver in 53.3% of the cases. Other types of nodules (FNH and hemangioma) were associated in 22.2% of cases. NTL was globally normal (Figure 2(d)). 

**Figure 4:**
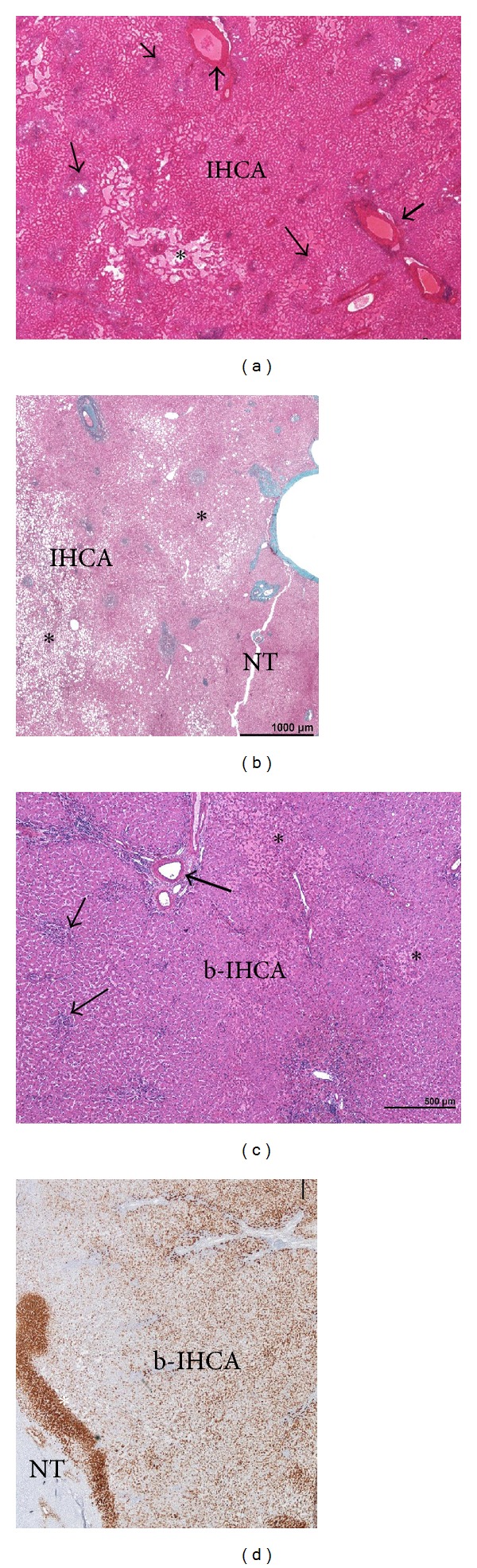
(a-b): Inflammatory (IHCA)—(a) typical aspect of IHCA with dispersed inflammatory foci (thin arrow), thick arteries (thick arrow), and areas of sinusoidal dilatation/peliosis (asterisk); (b) this nodule contains irregular areas of steatosis (asterisk); the limits of the tumor (IHCA) from the non tumoral liver (NT) are not visible on standard staining. (c-d) **β*-catenin activated inflammatory HCA (b-IHCA)*—(c) typical aspect of IHCA on H&E with inflammatory foci (thin arrow), thick arteries (thick arrow), and areas of sinusoidal dilatation (asterisk); (d) patchy positivity of glutamine synthetase immunostaining in tumor (b-IHCA), contrasting with nontumoral liver (NT).

*IHCA.* This group included 44 patients (39 women and 5 men) (Figures 1(a)–1(e)). The median age was 41.5; 92.3 of women were under OC. The median size of the largest nodule was 6 cm, and nodules were solitary in 63.6%. 54.5% of the patients have raised BMI (29.5% > 30). BMI scoring revealed a strong association between IHCA and high BMI score (Figure 1(a), *P* < 0.05). CRP and GGT were raised in 68.2 and 75%, respectively; AP, GGT, and CRP were higher than in the other HCA groups (H-HCA/b-HCA/UHCA (*P* < 0.01). The hallmark of IHCA was the inflammation (presence of inflammatory cells mainly around pseudoportal tracts, sometimes in foci dispersed inside the tumor) and pseudoportal tracts (with thick-walled arteries) present in 97.7 and 90.9%, respectively, (Figure 2(b)), as well as sinusoidal dilatation, which was major or moderate in 30 and 47.5% respectively but focal in 57.5% (Figures 2(c) and 4(a)). Steatosis was present in 38.6% of cases but focal in 88.2% and moderate in 47% (Figures 2(a) and 4(b)). Micro-IHCA were found in 36.4% in surrounding liver of the resected specimen. Surprisingly, in 3 cases, micro H-HCA (<5 mm, solitary or multiple in 2 or 1 cases, resp.) confirmed by lack of LFABP expression was incidentally observed on the surgical specimen at distance of IHCA. Other types of nodules (FNH and hemangioma) were present in 9.1%. The non tumoral liver was steatotic in 27%, and mild sinusoidal dilatation was observed in 13.6%; both steatosis and sinusoidal dilatation were more frequently observed in IHCA group in comparison to others (Figure 2(c), *P* < 0.001). 

Comparison between the H-HCA and IHCA groups showed that multiple nodules were more frequently observed in the H-HCA group than in the IHCA group (*P* < 0.05, Figure 2(d)). It is important to note that inflammatory cells, pseudo-PT, or ductular reaction were rare, often not detected in other HCA subgroups than IHCA (Figure 2(a)). However, due to the low number of patients in b-IHCA group, no significance difference was observed when compared to H-HCA group. 

In summary, considering standard histological features, steatosis (frequency and area) and sinusoidal dilatation (frequency and area) may represent the hallmark of H-HCA and IHCA, respectively, (Figure 2, *P* < 0.05 and *P* < 0.01, resp.).

*b-IHCA.* This group included 9 patients (3 men) and represented 17% of all IHCAs. Clinical abnormalities similar to that observed in IHCA were found (Figure 1). The standard pathological data were identical to IHCA (Table 1, Figure 4(c)), and therefore b-IHCA could not be identified as such using standard stainings; however, abnormal GS staining helps for the right diagnosis (Figure 4(d)).

#### 3.1.2. Group B

*Other Subtypes.* It included 16 patients, all women (4 b-HCAs and 12 UHCAs, median age 26 and 26.5, resp.). b-HCA subtype was more frequently detected in young patients (age < 25; *P* < 0.01, Figure 1(d)) than in other HCAs subtypes. Neither specific pathological features nor cytological abnormalities were observed. Areas with many thin dilated veins were observed in 3 cases. In all these 4 b-HCA cases, GS staining was patchy, as defined above (Figure 4(d)).

**Figure 5:**
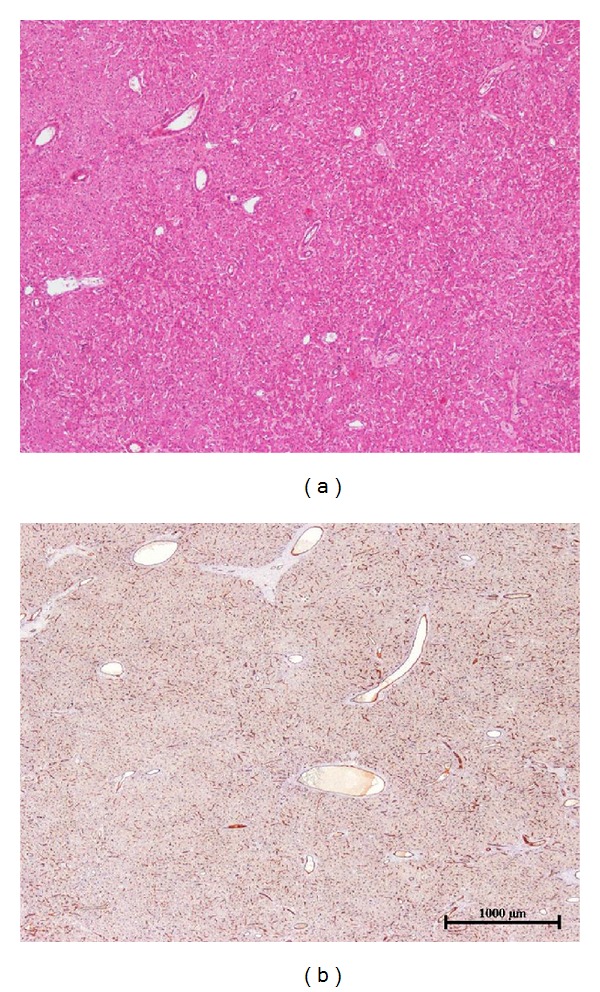
Unclassified HCA (UHCA)—(a): this nodule does not exhibit particular features on H&E; (b): nearly diffuse positivity of CD34 immunostaining.

In UHCA, the median age was 32.9. In half cases, UHCA occurred in young women (mean age 24.5). 50% were overweight/obese. UHCAs were detected often during complications (hemorrhage and necrosis) associated with large tumors. No specific pathological features usually existed (Figure 5(a)), but focal steatosis and sinusoidal dilatation were observed in 16.7 and 25%, respectively (Table 2). In 2 cases, numerous thin dilated veins were focally observed. In 2 cases, a few pseudo glandular arrangements were noticed. In 6 cases, tumoral nodules diffusely expressed CD34 (Figure 5(b)). In all cases, GS staining was normal. 

### 3.2. External Validation: Identification of HCA Subtypes from the Cohort (91 Cases)

#### 3.2.1. Group A

Among the 45 H-HCA, 40 were classified correctly (H-HCA positive and IHCA negative). There were 3 false negative readings (H-HCA negative and IHCA +/or possible/or negative. There were 2 cases with no formal diagnosis (H-HCA possible and IHCA possible). In 3 cases correctly classified H-HCA, GS was abnormal, with mild and patchy staining irregularly distributed within the tumor.

Among the 27 IHCAs, 24 were correctly classified, as well as 4 out of the 10 b-IHCAs. Interestingly enough, none of the b-IHCAs were identified without GS. There were 3 false negative readings: 1 in the IHCA and 2 in the b-IHCA. There were 6 cases with no formal diagnosis (1 in IHCA and 4 in the b-IHCA series. Among the 27 IHCAs, GS was abnormal in 1 case.

#### 3.2.2. Group B (5 b-HCAs and 4 UHCAs)

Among the 9 patients, 8 were correctly classified. One UHCA case was classified IHCA.

The agreement between routine technics combined to GS compared to the all sets of IHC markers for the identification of the 2 major HCA subtypes gave a kappa index of 0.89, 0.91/0.54 (group A), and 0.92 (group B) (*P* < 0.01) for H-HCA, IHCA/b-IHCA, and b-HCA/UHCA respectively, indicating a good agreement between them.

## 4. Discussion

Classification of HCA subtypes should allow the selection of patients at high risk of malignant transformation [7–10]. Specific immunohistochemistry is regarded as a main tool to classify HCA in clinical practice [2, 11, 12] and still remains the best method to make the differential diagnosis between FNH and HCA [5, 6]; however, it may not be widely available due to the fact that HCA is a rare and essentially benign disease.

The present study demonstrates the value of routine histology to classify the 2 major HCA subtypes: H-HCA and IHCA (Table 2), which represents more than 80% of cases at least in Europe, without the use of specific IHC. The diagnosis of H-HCA was often easy on routine H&E due to the presence of fat, a sign often used on MRI to identify this subtype [13–16]. The major criterion to identify IHCA was inflammation associated with pseudo-portal tracts containing thick-walled arteries, with frequent ductular reaction [5, 6]. Sinusoidal dilatation, a major criteria used by radiologist to identify IHCA was less sensitive than inflammation to diagnose IHCA. Interestingly enough, it was not possible to identify b-IHCA in the absence of GS staining. Overall, it was possible to strongly suspect an H-HCA and IHCA/b-IHCA on standard routine staining combined to GS in more than 90% of cases related to their hallmark features (Figures 3(a) and 4(a)) and by default the group b-HCA/UHCA. Interestingly enough, it was also possible to distinguish b-HCA from UHCA. 

Moreover, this study outlines the limits of routine histology to identify with certainty H-HCA and IHCA (including b-IHCA). First, in H-HCA, steatosis can be mild, focal, or rarely absent, and furthermore areas of sinusoidal dilatation can exist. On the other hand, in IHCA and b-IHCA, sinusoidal dilatation can be mild, focal, or absent; inflammation limited to few portal tracts-like and steatosis—generally focal—could be severe. In addition, in these 2 main subgroups, as well as in all cases of HCA, necrotic areas may lead to remodeling and more or less misleading features. In IHCA particularly, fibrotic bands associated with inflammation and ductular reaction may mimic FNH on routine histology. Additional IHC is mandatory to assess the right diagnosis. The fact that in the control set, we missed 6 out of 10 b-IHCAs based on IHCA criteria raises the question of the similarity between the 2 entities. The number of b-IHCA was, however, too small to be entirely sure to draw any firm conclusion.

If routine histology represents a reasonable means to identify the 2 major subtypes for pathologists with no access to specific IHC, the lack of identification of b-IHCA remains a serious limitation. Interestingly enough, this study demonstrates the impossibility to identify b-IHCA using standard histology. Surprisingly, none of the b-IHCA in our series (test and validation) had cytological or architectural abnormalities such as rosette formation as previously mentioned in b-HCA [3]. Abnormal GS expression is a very useful marker to orientate towards *β*-catenin-activated HCA (inflammatory or not), particularly when the staining is strong, diffuse, or patchy, even in the absence of aberrant *β*-catenin nuclear staining. However, GS reading is not always easy as underlined above, particularly when GS staining is faint and focal; in these cases, the distinction between a truly abnormal staining and, a few positive cells around veins in the nodule is hard to make. Indeed, this was the case in rare H-HCA. Cases of *β*-catenin activation could be observed in H-HCA, but this seems extremely rare. Therefore, the main issue remains the differential diagnosis between IHCA and b-IHCA. In these difficult cases, if there is no nuclear *β*-catenin staining, the issue can be solved only by molecular biology. 

More generally speaking, in the absence of specific complete set of IHC stainings, routine histology combined with GS can be considered as a reasonable surrogate approach for the identification of the 2 major HCA subgroups. In addition, clinical, biological, and radiological data can help to make the right diagnosis of HCA subtype [17, 18]. 

We have to remember that the very peculiar pattern of GS immunostaining in FNH is particularly helpful for their diagnosis mainly when different types of nodules are associated with the same liver. Finally, in the present work, we acknowledged that the H-HCA and IHCA/b-IHCA subtypes using routine histology were obtained on surgical specimens. In a recent article, we demonstrate that typical H-HCA can be recognized on biopsy on H&E staining; this is also possible for IHCA, but the % of correct diagnosis is lower [19]. Therefore, to avoid the difficulty of interpretation and the higher risk of errors on biopsies, IHC is highly recommended. IHC not only offers the possibility to identify HCA subtypes, but also to confidently make the differential diagnosis between FNH and HCA [19].

## 5. Conclusion

The diagnosis of HCA may be difficult particularly for the pathologists are not familiar with these rare tumors. Characteristic features may exist which can be recognized by general pathologists on routine histology, allowing a good estimation of the 2 major HCA subtypes (>80%). Therefore, a “palliative” diagnosis approach can be proposed, when the panel of the antibodies is not available. However, in absence of these features, IHC remains the gold standard to assess the diagnosis of HCA and its subtype. GS is essential to identify *β*-catenin-activated HCA (inflammatory or not) that is particularly important to detect patient with higher risk of malignant transformation.

## Supplementary Material

Supplementary Table 1: Pathological analysis of resected hepatocellular adenoma.
Supplementary Table 2: Clinical data of hepatocellular adenoma subtypes.

## Figures and Tables

**Table 1:** Pathological data of HCA subtypes.

	H-HCA (*n* = 45)	IHCA (*n* = 44)	b-IHCA (*n* = 9)	b-HCA (*n* = 4)	UHCA (*n* = 12)
Steatosis	43	17	1	1	2
>60%/30–60%/10–30%	11/19/13	1/8/8	0/0/1	1/0/0	1/1/0
Focal/spread/diffuse	9/22/12	15/2/0	1/0/0	1/0/0	2/0/0
Sinusoidal dilatation	11	40	9	0	3
Major/moderate/mild	0/4/7	12/19/9	3/3/3	0	0/1/2
Focal/spread/diffuse	10/1/0	23/12/5	8/1/0	0	2/1/0
Peliosis	9	10	5	0	3
Pseudo-PT	1	40	6	0	0
Inflammation	0	43	7	1	0
Ductular reaction	0	31	4	0	2
Remodeling	5	7	3	1	3
Cytological abnormalities	2	0	0	0	2
Micro-HCA*	24	16	1	0	0
Others types of nodules	10	4	1	1	0
NTL					
Steatosis	2	12	2	0	3
Sinusoidal dilatation	1	6	1	1	2
NASH	0	1	0	0	0
Fibrosis	0	1	2	0	1
Portal embolisation	0	4	1	1	0

HCA: hepatocellular adenoma; H-HCA: HNF1*α* mutated HCA; IHCA: inflammatory HCA; b-IHCA: *β*-catenin mutated IHCA; b-HCA: *β*-catenin mutated HCA; UHCA: unclassified HCA; PT: Portal Tract; *: at distance of main tumor; NASH: non alcoholic steatohepatitis; NTL: non tumoral liver.

**Table 2:** Phenotypic classification of hepatocellular adenoma: routine histology and immunohistochemistry (IHC) and molecular biology.

Routine histology	In favor of	IHC
(i) Diffuse steatosis (ii) Lobulated contour (surgical specimen) (iii) No criteria for IHCA	H-HCA	Lack of LFABP → H-HCA
(iv) Inflammation (v) Sinusoidal dilatation (vi) Pseudoportal tracts (with thick arteries) (vii) Ductular reaction	IHCA*	CRP/or SAA + → IHCA CRP/or SAA +/GS**+ → b-IHCA
		GS** + (CRP−) → b-HCA
		All markers− → UHCA

In the absence of typical routine histological criteria for H-HCA or IHCA, the other HCA subtypes are likely *include b-IHCA (GS is mandatory to differentiate IHCA and b-IHCA).

**Perform in addition *β*-catenin staining: aberrant nuclear staining confirm the diagnosis; its absence does not rule out; however, the diagnosis, particularly on needle biopsies, needs molecular biology for definite diagnosis.
