# Supplementary material for: Value and Limits of Routine Histology Alone or Combined with Glutamine Synthetase Immunostaining in the Diagnosis of Hepatocellular Adenoma Subtypes on Surgical Specimens
Source: Int J Hepatol. 2013 Feb 19;2013:417323. doi: 10.1155/2013/417323 (PMC3590632; doi:10.1155/2013/417323)
Supplement: Supplementary file 1 — Supplementary Table 1: Pathological analysis of resected hepatocellular adenoma. Supplementary Table 2: Clinical data of hepatocellular adenoma subtypes. [file 417323.f1.docx]

**Pathological analysis of resected HCA**

Code investigator date circle the appropriate response

**Macro**  size cm color % necrosis % hemorrhage /

**Micro**

1 Steatosis: **Yes**  1 2 3 **No**  (1: major >60%, 2: moderate 30-60%, 3: mild 10-30%)

a: focal; b: spread, c: diffuse

2 Sinusoidal dilatation **Ye**s 1 2 3 **No** Congestion **Yes**  1 2 3 **No** Peliosis **Yes** 1 2 3 **No** (1: major, 2: moderate, 3: mild) a: focal; b: spread, c: diffuse

3 Pseudo PT **Yes** 1 2 3 **No** ( 1: many, 2: some, 3: few)

4 Thick-walled arteries : arteries in a fibrous atmosphere/isolated **Yes** 1 2 **No** (1: major, 2: minor)

5 Inflammation **Yes** 1 2 3 **No** ( 1: major, 2: moderate, 3: mild )

6 Ductular reaction **Yes** 1 2 3 **No** ( 1: major, 2: moderate, 3: mild)

7 Areas of necrosis (micro) **Yes**  1 2 **No** ( 1: major, 2: minor)

8 Areas of hemorrhage (micro) **Yes**  1 2 **No** (1: major, 2: minor)

9 Remodeling fibrotic bands **Yes** 1 2 **No** (1 many, 2: some)

10 cytological abnormalities (rosettes) **Yes** **No**; areas of dysplasia **Yes** **No** ; HCC **Yes No**

11 micro-HCA **Yes** : histo/ IHC 1 2 **No** (1 many, 2 few)

**Non tumoral liver:** absent; border (1 cm or less); present (<2 cm; > 2 cm)

normal ; steatosis 1 2 3 (idem above); sinusoidal dilatation (1 2 3) (idem above)

NASH **Yes No**; vascular pathology

IHC: **glutamine synthetase** (GS)

a- normal staining (absent or present essentially around veins/border)

b- abnormal : GS strong, moderate, weak: diffuse or focal; GS patchy : diffuse or focal

c- GS difficult to interpret (faint, focal)

IHC **: LFABP**

a absent T/ present NT (no NT) ; b no major difference between T (weak stain) and NT (NT absent)

IHC: **SAA /CRP/both**

+ T: strong/ moderate/weak/diffuse/heterogeneous / - NT / + NT weak/ strong / no NT

IHC b cat

Nuclear staining **yes** 1 nucleus, some, many **No**
