# Supplementary material for: Value and Limits of Routine Histology Alone or Combined with Glutamine Synthetase Immunostaining in the Diagnosis of Hepatocellular Adenoma Subtypes on Surgical Specimens
Source: Int J Hepatol. 2013 Feb 19;2013:417323. doi: 10.1155/2013/417323 (PMC3590632; doi:10.1155/2013/417323)
Supplement: Supplementary file 2 [file 417323.f2.docx]

Supplemental Table 2 - Clinical data of HCA subtypes

		H-HCA	IHCA	b-IHCA	b-HCA	UHCA
**n**		45	44	9	4	12
**Age : median (extremes)**		40 (23 ; 60)	41.5 (25 ; 59)	36 (26 ; 59)	26 (21 ; 29)	26.5 (21 ; 48)
**Sex (F ; M)**		45 ; 0	39 ; 5	6 ; 3	4 ; 0	12 ; 0
**BMI**	**BMI ≥ 30**	3 / 44	13	1	0	2
	**BMI ≥ 25**	8 / 44	11	2	0	4
	**BMI < 25**	33 / 44	20	6	4	6
**OC**		38	36	5	4	12
**Imaging**	**single nodule**	15	28	6	4	9
	**multiple nodules**	28	16	3	0	3
	**Size of the largest resected nodule median (extremes)**	5.6 (1.5 ; 15)	6 (1 ; 14.5)	7 (3 ; 19)	8.3 (5 ; 14)	7.5 (3 ; 14)
**Elevated enzymes**	**CRP**	4	30	4	0	8
	**AP**	2	20	3	0	0
	**GGT**	10	33	5	2	8

HCA : hepatocellular adenoma ; H-HCA : HNF1α mutated HCA ; IHCA : inflammatory HCA ; b-IHCA: β-catenin mutated IHCA ; b-HCA : β-catenin mutated HCA ; UHCA : unclassified HCA ; BMI : Body Mass Index ; OC : Oral Contraceptive ; CRP : C Reactive Protein ; AP : Alkalin Phosphatase ; GGT : Gamma Glutamyl Transferase
